# Ultrasound-based liver elastography: current results and future perspectives

**DOI:** 10.1007/s00261-020-02717-x

**Published:** 2020-09-11

**Authors:** Cheng Fang, Paul S. Sidhu

**Affiliations:** Department of Radiology, King’s College Hospital, Denmark Hill, London, SE5 9RS UK

**Keywords:** Liver elastography, Transient elastography, Fibrosis, Hepatitis, Cirrhosis, Chronic liver disease

## Abstract

Chronic liver disease affects 185 million population worldwide. It encompasses a heterogenous disease spectrum, but all can lead to the development of liver fibrosis. The degree of liver fibrosis is not only a prognosticator, but has also been used to guide the treatment strategy and to evaluate treatment response. Traditionally, staging of liver fibrosis is determined on histological analysis using samples obtained from an invasive liver biopsy. Ultrasound-based liver elastography is a non-invasive method of assessing diffuse liver disease in patients with known chronic liver disease. The use of liver elastography has led to a significant reduction in the number of liver biopsies performed to assess the severity of liver fibrosis and a liver biopsy is now reserved for only select sub-groups of patients. The aim of this review article is to discuss the key findings and current evidence for ultrasound-based elastography in diffuse liver disease as well as the technical challenges and to evaluate the potential research direction.

## Introduction

Chronic liver disease affects 185 million people worldwide [1]. It encompasses many disease aetiologies with the vast majority of cases secondary to viral hepatitis, alcohol induced, non-alcoholic fatty liver disease (NAFLD), non-alcoholic steatohepatitis (NASH), autoimmune hepatitis (AIH). The common pathological consequence for all these different aetiologies involves the development of liver fibrosis; a result of chronic inflammation. Untreated, liver fibrosis can lead to end stage liver disease and cirrhosis both of which are associated with a high morbidity and mortality. Assessment of the degree of liver fibrosis is clinically relevant in guiding treatment, evaluating response and predicting associated complications. Ultrasound-based elastography is an adjunctive tool to traditional B-mode ultrasound for assessing patients with chronic liver disease. It is inexpensive, quick to perform and has a short period of training for the operator.

Liver elastography guidelines are available from European Federation of Societies for Ultrasound in Medicine and Biology (EFSUMB) [2], World Federation for Ultrasound in Medicine and Biology (WFUMB) [3, 4] and Society of Radiologists in Ultrasound Consensus [5]. The aim of this review article is to discuss the key findings and current evidence for ultrasound-based elastography in diffuse liver disease as well as technical challenges and potential further research.

## Types of elastography

Elastography is a method of studying tissue stiffness. The differences in tissue stiffness between healthy and pathological tissue allows for the inference of the presence and severity of disease. There are two types of ultrasound-based elastography techniques; strain elastography (SE) and shear wave elastography (SWE).

### Strain elastography

**Fig. 1:**
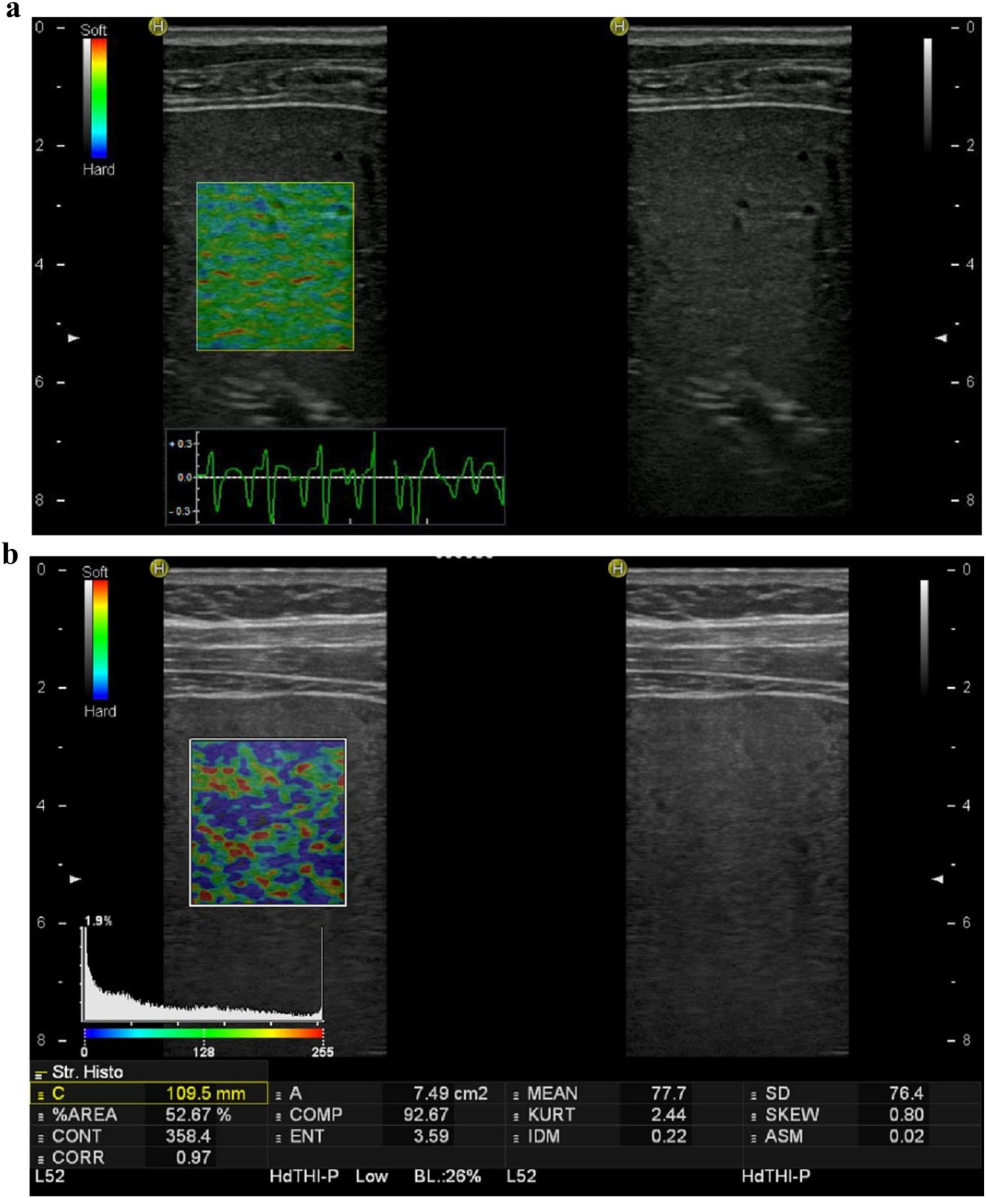
Two strain elastography studies performed using Real-time Tissue Elastography (Hitachi Medical Corporation, Japan). **a** Liver elastogram from a 21-year-old male with chronic hepatitis B infection demonstrates “soft” liver texture indicated by relatively homogenous green colour. **b** Liver elastogram from a 59-year-old male with chronic hepatitis C infection demonstrates “hard” liver texture which corresponding to severe fibrosis on histological analysis. The parameters underneath the pictures can be used to compute semi-quantitative liver fibrosis index

In SE the elasticity of the tissue of interest is assessed by comparing the degree of distortion with adjacent healthy tissue induced by an external manual compression or cardiac pulsation [6, 7]. The output is a colour coded map of tissue elastogram super-imposed on the B-mode image (Fig. 1). This shows the relative degree of distortion between the tissue of interest and adjacent tissue. Therefore, the stain ratio between the pathological and healthy tissue is a relative measure of tissue elasticity.

Strain elastography was first available commercially on the Hitachi platform also known as Real-time Tissue Elastography (RTE™, Hitachi Inc, Japan). Semi-quantitative analysis can be performed by calculating the liver fibrosis index (LFI) from 11 imaging features including mean relative strain value (MEAN), standard deviation of relative strain value (SD), percentage of lower strain area (% of blue colour area − % area), complexity of lower strain area, Skewness (SKEW), Kurtosis (KURT), Entropy (ENT), textural complexity, inverse difference moment (IDM), angular second moment, contrast (ASM), and correlation (COR) [8] (Fig. 1). The results from a RTE™ study is more operator-dependent and requires additional training when compared to using the SWE techniques [9]. There have been far fewer publications using SE compared to SWE and these studies were predominantly performed in East Asian populations, where obesity is less prevalent. Meta-analysis showed the AUROC for significant fibrosis (F > 2), advanced fibrosis (F > 3) and cirrhosis (F = 4) were 0.79, 0.94 and 0.85 using LFI [10]. Furthermore, few studies have compared diagnostic performance of RTE™ against SWE [9, 11]. The use of RTE™ in assessing stages of liver fibrosis has not been incorporated in EASL, EFSUMB [6], SRU [5] and WFUMB guidelines [3, 4].

Assessment of liver stiffness using both SWE (2D-SWE) and SE can be simultaneously performed on ARIETTA Hitachi 850 known as Combi-Elasto (Hitachi Medical Corporation). Assessing liver stiffness using combined RTE™ and the shear wave technique could potentially identify the influence of any confounding factors on liver stiffness such as acute inflammation, biliary obstruction or hepatic congestion. During follow-up studies changes in relative strain during RTE™ examination is thought to be rarely affected by these factors [9].

### Shear wave elastography

In SWE, an acoustic impulse is generated by the transducer and transmitted from the transducer to the region of interest (ROI) where the propagation speed of the resultant shear wave is measured. Tissue elastic modulus is calculated through the equation, *E* = 3*ρc*^2^, where *E* is tissue elasticity, *C* is shear wave velocity, and *ρ* is the density of the tissue in kg/m^3^. Therefore, shear wave velocity is a quantitative method of determining the absolute tissue elasticity. Shear wave elastography encompasses several different methods of measuring shear wave velocities: transient elastography (TE), point shear wave elastography (pSWE) and two-dimensional shear wave elastography (2D-SWE). There are many commercially available machine platforms allowing users to perform a liver elastography study are listed, but not exclusively, in Table 1.

**Table 1:** Examples of current available commercial systems (applications)

Commercially available systems	Software name	Reliability indication
*Transient elastography*		
Fibroscan™ (Echosens, France)	Fibroscan™	IQR/M ≤ 30%
*Point shear wave elastography*		
ACUSON™ (S2000/S3000/Sequoia), (Siemens Healthineers, Germany)	Virtual Touch™ Quantification (VTQ™)	IQR/M ≤ 30%
EPIQ series, Affinity—(Philips Healthcare, Netherlands)	ElastPQ™	IQR/M ≤ 30%
Ascendus™, Arietta series—(Hitachi Medical Corporation, Japan)	Hi-VISION™	Net amount of effective shear wave velocity percentage (VsN) ≥ 50%
MyLab™ Twice (Esaote SpA, Italy)	QElaXto	IQR/M ≤ 30%
RS80, HS70A (Samsung Medison, South Korea)	S-Shearwave	Reliable measurement index (RMI) > 80%
*2D*-*shear weave elastography*		
Aixplorer™—(Aix-en-Provence, France)	SuperSonic Imaging (SSI™)	Stability index
LOGIQ (E9, E10)—(GE Healthcare, USA)	2D comb-push	IQR/M ≤ 30%
ACUSON™ (Sequoia), (Siemens Healthineers, Germany)	Virtual Touch™ Quantification (VTQ™)	IQR/M ≤ 30%
MyLab™ 9eXP (Esaote SpA, Italy)	QElaXto -2D	Quality colour map
RS85—(Samsung Medison, South Korea)	S-Shearwave	Reliable measurement index (RMI) > 80%
Aplio 500™—(Toshiba, Japan)	Acoustic Structure Quantification (ASQ)	Shear Wave Propagation map
EPIQ series—(Philips Healthcare, Netherlands)	ElastPQ™ imaging	Confidence map

TE is more commonly known by the brand name FibroScan™ (Echosens, Paris, France). TE displays shear wave velocity by converting this to kilopascals (kPa) but does not have the capability of forming a B-mode image of the liver during the elastography examination. This cannot be used in patients with abdominal ascites as measurements will be unreliable and has technical limitations with obese patients. This may be overcome by using a different transducer, the XL transducer, which is designed for obese patients.

pSWE and 2D-SWE are performed with conventional ultrasound machines which allows simultaneous B-mode imaging allowing for a comprehensive liver assessment. The difference between pSWE and 2D-SWE is that the former emits a single shear wave at a single frequency for each measurement, while the latter emits multiple shear waves simultaneously. Conventionally the measurements are expressed as a velocity, although many manufacturers are able to also display measurements in kPa. Conversion from velocity measurements to kPa results in additional inaccuracies but TE and FibroScan™ have established the kPa as a readily identifiable measurement in hepatology practice.

## Elastography examination techniques

Patients are required to fast for at least a minimum of 2 h and rest for 10 min prior to the examination to avoid falsely elevated liver stiffness measurements [12]. The examinations are performed with patients lying in supine position with their right arm extended over their head to increase intercostal space. Measurement should be taken from the right lobe of the liver via intercostal costal approach. Using the FibroScan™ technique, there is no region of interest to place as there is no visualization of the liver, and readings are obtained from the perceived correct area of liver. With ultrasound-based techniques, where the liver is visualized, operators should place the region of interest (ROI) box in the right lobe of the liver avoiding any rib shadows, large blood vessels and the biliary tree. There are a number of commercial systems which incorporate both pSWE and 2D-SWE examinations within the machine functionality, as summarized in a recent review [13].

**Fig. 2:**
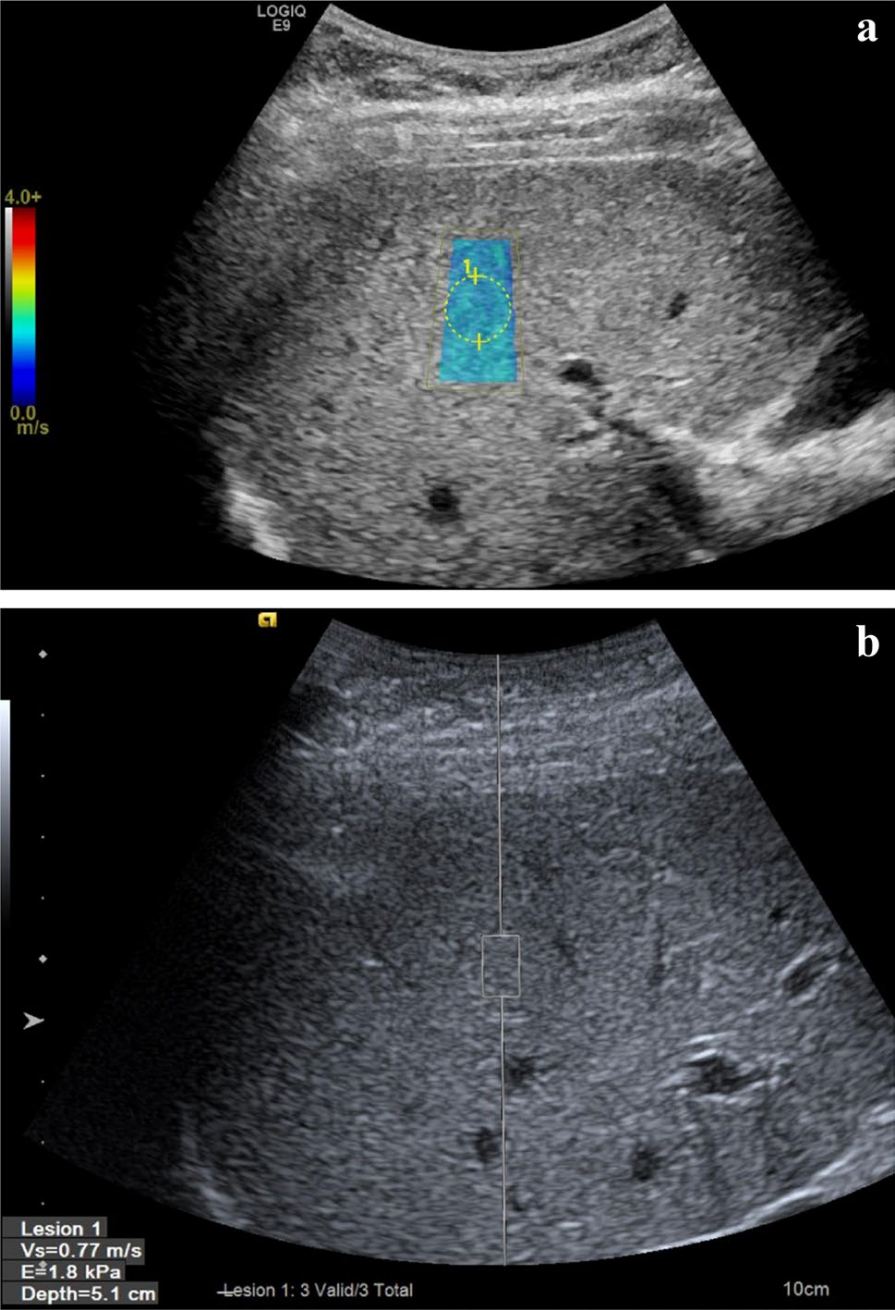
Comparative pSWE and 2D-shear wave studies from the same patient. 44-years-old female with chronic hepatitis. **a** 2D-SWE performed on GE (LOGIQ E9) showed normal shear wave velocity indicated by homogenous blue colour. The dotted ROI circle was placed to calculate the velocity which is 0.8 m/s. **b** pSWE performed on Siemens (S3000 Acuson). A colour map was not produced using this technique instead the average shear wave velocity (0.77 m/s) within the rectangular ROI box was displayed in the left bottom corner on the screen. Patient had same day liver biopsy and showed to have Ishak fibrosis score of 0 from histological analysis

**Fig. 3:**
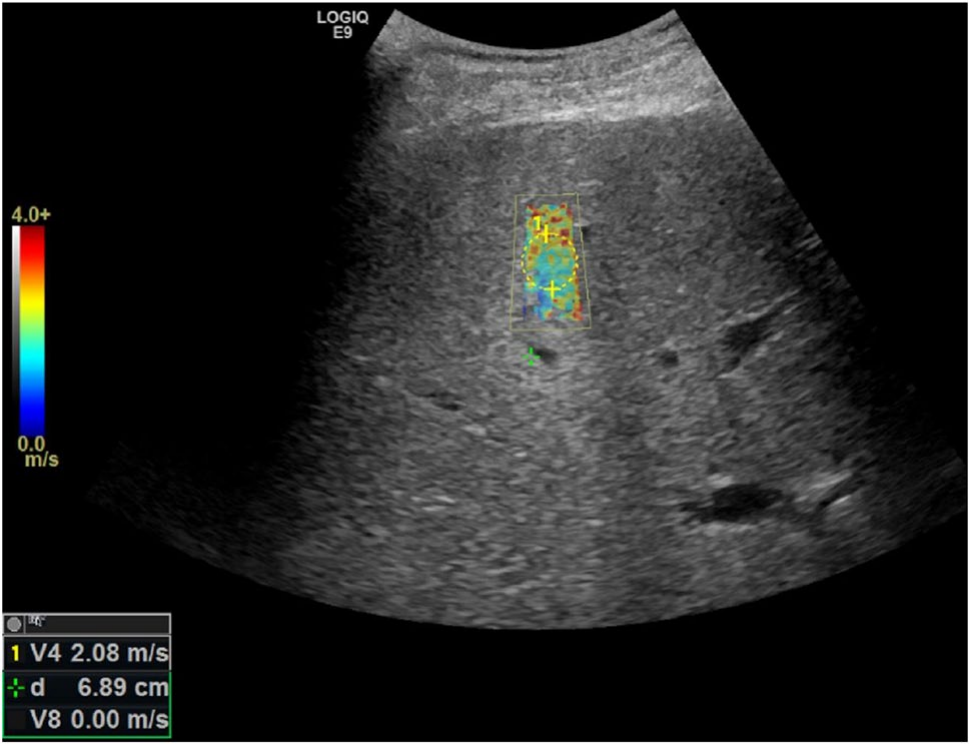
50-year-old male with alcohol-related liver disease. 2D-SWE (GE) showed heterogeneous mixed green, yellow and red colour elastogram box. The centre round dotted region of interest box was placed to calculate the shear wave velocity after the images were acquired

For pSWE examination, the ROI box should be placed 1–2 cm below the liver capsule to avoid reverberation artefact and the subcapsular liver parenchyma which is stiffer, a consequence of the proximity to the liver capsule tissue (Fig. 2). This is not strictly necessary for 2D-SWE, as a colour map of tissue elastogram will be displayed for the ROI box and subsequent separate analysis box can be placed within the ROI box, avoiding any reverberation artefact (Fig. 3). Additionally, for both pSWE and 2D-SWE, the transducer should be perpendicular to the liver capsule.

Using the median of 10 measurements as the stiffness value was recommended as standard practice when using the FibroScan™ machine and has subsequently been adopted as the required number of measurements in clinical practice by majority of pSWE studies, and has become the accepted requisite number of measurements supported by both the EFSUMB and WFUMB guidelines [4, 14]. However, recent evidence has suggested that fewer measurements may be acceptable when using pSWE [15]. For 2D-SWE, the availability of the colour stiffness map prior to selecting the analysis box reduces the data variability allowing few measurements [16]. EFSUMB and WFSUMB both suggested that a minimum of three measurements be taken [4, 14] and recent meta-analysis from 34 2D-SWE studies showed fewer unreliable measurements in those studies which obtained more than three measurements [17]. All guidelines recommend the use of interquartile range over median (IQR/M), an indicator for data variability, to assess data quality when performing liver stiffness measurement using TE and pSWE techniques. Liver stiffness values with IQR/M of ≤ 30% are associated with increased accuracy for staging liver fibrosis [15, 18–20] as well as has a better agreement among different elastography machines [21]. Reducing the number of liver stiffness measurements has also been shown to have little effect on diagnostic accuracy using measurements with IQR/M ≤ 30% [15]. However, IQR/M can only be obtained once the study has been completed and more advanced stages of fibrosis can also influence the data variability, hence affecting the IQR/M. Newer systems have incorporated reliability indicators for each measurement displayed at the time of study, which allows the examiner assess the data reliability at the time of the examination [22–24].

##  Normal values of shear wave velocities and potential confounding factors

Current literature for liver stiffness measurement in healthy volunteers is summarized by Dong et al. [25]. Among the larger studies, the mean/median liver stiffness value using FibroScan™ ranges between 4.1 to 5.5 kPa with calculated highest upper 95% percentile of 8.7 kPa [26–32]. The mean or median shear wave velocity measurements using pSWE are available for most commercial systems VTQ™ [33–37], ElastPQ™ [38, 39], Samsung RS80A [40] and these measurements are between 1.03 to 1.19 m/s, with the calculated highest upper 95% percentile value of 1.69 m/s and mean/median value of 4.95 kPa to 5.5 kPa with 95% percentile value of 8.04 kPa [41–45].

Liver elastography measures tissue stiffness which can be increased in circumstances other than the presence of liver fibrosis. There are known confounding factors which include post prandial status [46], physical exercise [12], any liver disorders causing acute inflammation associated with a transaminase rise [47], right heart failure causing liver congestion [48], the Valsalva manoeuvre during the examination and biliary obstruction [49]. Age, gender and body mass index are not thought to influence the liver stiffness value. The impact of hepatic steatosis on liver stiffness is uncertain. Although steatosis has not been proven to affect liver stiffness values, it will attenuate the shear wave.

## Performance of shear wave elastography

### Staging of fibrosis in chronic liver disease

SWE has been widely used to assess the degree of liver fibrosis in the management of patients with chronic liver disease in the last 5 years. In many centres, it has gradually replaced the “gold standard” of liver biopsy. In comparison to liver biopsy, liver elastography is non-invasive, has high patient compliance, good intra- and interobserver reproducibility [17, 50–55], whereas a liver biopsy is invasive with significant mortality and mobility [56, 57]. A liver biopsy is prone to sampling errors and the histological analysis has a high observer variability [58, 59].

The European Association for the Study of the Liver (EASL-ALEH) recommend TE as part of the non-invasive tests for evaluating severity and prognosis of liver disease by determining the stage of liver fibrosis [60]. In the United Kingdom, the National Health Service Institute for Health and Care Excellence (NICE) have also recommended the use of VTQ™ (pSWE, Siemens Healthineers, Germany) to assess liver fibrosis in patients with viral hepatitis B and C [61]. A number of meta-analyses have shown good to excellent diagnostic accuracy using TE in determining significant and severe liver fibrosis when the results were correlated with pathological classification of liver fibrosis with Area Under the Curve Receiver Operating Characteristic (AUROC) of 0.82–0.88 for significant fibrosis and 0.91–0.93 for severe fibrosis [62–64]. Similar AUROC values for significant fibrosis and severe fibrosis using pSWE (VTQ™) (0.88—significant fibrosis; 0.91—severe fibrosis) [65] and 2D-SWE AUROC (0.91—significant fibrosis; 0.95—severe fibrosis) [66] were reported. Both pSWE and 2D-SWE techniques have persistently demonstrated a higher technical success rate compared with TE [67, 68]. This is likely due to the ability to visualize the liver with the B-mode ultrasound component and place a region of interest within the liver, avoiding vascular/biliary structures, at the optimal depth during the pSWE and 2D-SWE examinations. Higher diagnostic accuracy has been reported for 2D-SWE compared to TE for all aetiologies of liver disease [69]. There are very few studies which have compared the diagnostic performance between 2D-SWE and pSWE; one early study showed a higher diagnostic performance using 2D-SWE in predicting significant fibrosis than with pSWE (VTQ™) [70] and later studies have showed similar performances [71, 72].

According to the EFSUMB guidelines, the optimal cut-off values for predicting significant fibrosis and cirrhosis in the presence of viral hepatitis C, for VTQ™ is 1.21 m/s–1.34 m/s and 1.55 m/s and 2.00 m/s. In the presence of viral hepatitis B, a meta-analysis has indicated that the optimal cut-off for predicting significant fibrosis and cirrhosis is 1.35 m/s and 1.87 m/s respectively [73]. The optimal cut-off measurement for a mixed aetiology is reported in a meta-analysis to be 1.30 ± 0.07 m/s for predicting significant fibrosis and 1.80 ± 0.16 m/s for cirrhosis [74].

### Measurement variation

More advanced stages of fibrosis will result in higher shear wave velocities, and this has been demonstrated in numerous studies. Whist these shear wave velocities differ significantly between the METAVIR classification categories of F1 and F4 liver fibrosis, the range of shear wave velocities can overlap significantly among the early and intermediate stages of fibrosis (F1 to F3). It has been also been observed that individual cut-off values for different fibrosis stages may also vary depending on disease aetiology and significantly, machine manufacturer, as absolute shear wave velocities measurements are not transferable between different machines [21, 52, 75–77]. This is an issue associated with the technique of measurement and calculation with each manufacturer processing the data obtained differently. This limitation may be overcome by using the same machine for all studies, but this may not be practicable. WFUMB has recommended applying a ‘rule of 5’ when interpreting liver stiffness values and managing patients; measurement < 5 kPa is normal, value between 5 and 10 kPa rules out compensated advanced chronic liver disease in the absence of known clinical signs, values between 10 and 15 kPa rules out advanced compensated chronic liver disease, values between 15 and 20 kPa highly suggests compensated advanced chronic liver disease and a value exceeding 20 kPa is in keeping with clinically significant portal hypertension [4]. This emphasizes the importance of interpreting liver stiffness measurement by liver specialists in conjunction with patient’s other clinical data rather than being used as a screening tool in the general population [60].

### Anti-viral therapy

There are increasing numbers of hepatitis C patients with a sustained virological response when treated with the new generation of anti-viral therapy [78]. Although histology regression of fibrosis has been demonstrated [79], the role and timing of liver elastography in assessing regression of liver fibrosis is yet to be established. This is because the established cut-off values may not be applicable following eradication of viral hepatitis and it may be difficult to differentiate the reduction of liver stiffness as being due to reduction of inflammation or regression of fibrosis. Abrupt reduction in liver stiffness values assessed have been reported immediately after anti-viral treatment [80, 81].

### Non-alcoholic fatty liver disease

NAFLD is increasingly prevalent and is becoming the most common cause of chronic liver disease worldwide [82, 83] with non-alcoholic steatohepatitis (NASH) becoming the main indication for liver transplantation in the United States [84]. Liver fibrosis has been reported to be the strongest predictor for long term disease specific mortality [85]. The EASL-ALEH clinical guidelines recommend non-invasive screening for liver fibrosis in NAFLD patients [60] with liver biopsy reserved for patients at high risk of advanced fibrosis if the non-invasive tests were unable to exclude advanced fibrosis [86]. Diagnostic performance of using TE, pSWE and 2D-SWE have all been reported in the literature. A recent meta-analysis reported cut-off values for severe fibrosis ranging from 7.6 to 9 kPa using TE with sensitivity between 83 and 89% and specificity between 77 and 78% [87]. Unreliable liver stiffness values and increased technical failure rate in NAFLD patients appears to be associated with obesity and use of M transducer on the FibroScan™ machine, both of which increases the false positive rate, using the XL transducer can overcome these issues [88]. Fewer studies are available for pSWE and 2D-SWE [89–91]. A recent meta-analysis reported good and comparable diagnostic performance with AUROC between 0.86–0.95 and 0.85–0.94 for TE and pSWE respectively [68]. A single meta-analysis has reported a superior diagnostic accuracy using 2D-SWE than TE [87].

### Alcoholic liver disease

For patients with alcoholic liver disease, there is no consensus in terms of the optimum cut-off values for significant fibrosis, severe fibrosis and cirrhosis due to a wide range of cut-off values reported from TE studies [92–96]. There are a limited number of studies using pSWE and 2-SWE. Currently, WFUMB recommends the use of shear wave elastography (including TE, pSWE, 2D-SWE) in patients with alcoholic liver disease to rule out advanced disease [4], while EFSUMB only recommends the use of TE for this indication [14]. Significantly reduced liver stiffness values have been reported in patients who have stopped consuming alcohol and falsely elevated values are seen in patients with acute alcohol intoxication with increased transaminases, bilirubin or gamma-glutamyl transferase. It is suggested that the best time to assess liver fibrosis is after a period of abstinence [97].

### Assessment of portal hypertension

Patients with significant and severe portal hypertension are at increased risk of developing varices and consequently acute variceal bleeding. The gold standard of assessing the presence and severity portal hypertension is through an invasive angiographic technique; venous pressure gradient (HVPG) measurement with a cut-off value of HVPG ≥ 10 mmHg indicating clinical significant portal hypertension and a HVPG ≥ 12 mmHg indicating the potential for variceal bleeding [98, 99]. SWE has also been used as a non-invasive tool for assessing the severity of portal hypertension in patients with chronic liver disease. A meta-analysis has shown consistent evidence supporting the use of a liver stiffness value as a biomarker for clinically significant portal hypertension, although the optimal cut-off value calculated from different studies varies from 15 to 25 kPa [100]. A further meta-analysis showed 2D-SWE values of 14 kPa or less can rule out clinically significant portal hypertension in cirrhotic patients with a reported AUROC value of 0.88 and sensitivity value of 91%. However, this does not predict severe portal hypertension with the presence of varices needing treatment [101]. The current evidence suggests that liver stiffness values show a good correlation with HVPG up to 12 mmHg but cannot replace invasive assessment for assessing the severity and progression of the portal hypertension, primarily as the SWE values are less dependent on intrahepatic resistance with the development of fibrosis [60]. According to the Baveno VI consensus, patients with liver stiffness of < 20 kPa and platelet count of > 150,000 can avoid screening endoscopy for esophageal varices [102].

## Artificial intelligence

The use of artificial intelligence (machine learning) in analysing medical images has experienced an exponential growth in recent years due to the arrival of deep learning methods using convolutional neural networks (CNNs) [103] and its superior accuracy compared to traditional supervisor classification methods [104]. Most recently, Wang et al. showed superior diagnostic performance in predicting severe liver fibrosis (F ≥ 3) and cirrhosis (F4) in patients with chronic hepatitis B by analysing images from 2D-SWE studies using deep learning radiomics compared to 2D-shear wave velocity measurements [105]. Gatos et al. also demonstrated that by identifying areas of high and lower temporal stability from 2D-SWE images by means of deep learning algorithm, the diagnostic accuracy and inter observer variability are better when using areas of high temporal stability (reliable areas) than that from lower temporal stability (unreliable areas) [106].

## Conclusion

Ultrasound-based elastography offers a cost-effective, non-invasive and accurate method of assessing the severity of diffuse liver disease. Currently, it has superior accuracy in predicting cirrhosis rather than significant fibrosis. Its high negative predictive value should be incorporated into the decision-making process in managing patients with chronic liver disease. Individual optimal cut-off values of fibrosis stages for different aetiologies are yet to be validated in large prospective studies.

## References

[1] Byass P (2014) The global burden of liver disease: a challenge for methods and for public health. BMC Med 12: 10.1186/s12916-014-0159-5 10.1186/s12916-014-0159-5 PMC4168048 25286285

[2] Cosgrove D, Piscaglia F, Bamber J, et al (2013) EFSUMB Guidelines and Recommendations on the Clinical Use of Ultrasound Elastography. Part 2: Clinical Applications. Ultraschall in der Medizin - European Journal of Ultrasound 34:238–253. 10.1055/s-0033-1335375 10.1055/s-0033-1335375 23605169

[3] Ferraioli G, Filice C, Castera L, et al (2015) WFUMB guidelines and recommendations for clinical use of ultrasound elastography: Part 3: liver. Ultrasound Med Biol 41:1161–1179. 10.1016/j.ultrasmedbio.2015.03.007 10.1016/j.ultrasmedbio.2015.03.007 25800942

[4] Ferraioli G, Wong VW-S, Castera L, et al (2018) Liver Ultrasound Elastography: An Update to the World Federation for Ultrasound in Medicine and Biology Guidelines and Recommendations. Ultrasound in Medicine & Biology. 10.1016/j.ultrasmedbio.2018.07.008 10.1016/j.ultrasmedbio.2018.07.008 30209008

[5] Barr RG, Ferraioli G, Palmeri ML, et al (2016) Elastography Assessment of Liver Fibrosis: Society of Radiologists in Ultrasound Consensus Conference Statement. Ultrasound Q 32:94–107. 0.1097/RUQ.0000000000000209 10.1097/RUQ.0000000000000209 27233069

[6] Cosgrove D, Piscaglia F, Bamber J, et al (2013) EFSUMB Guidelines and Recommendations on the Clinical Use of Ultrasound Elastography.Part 2: Clinical Applications. Ultraschall in der Medizin - European Journal of Ultrasound 34:238–253. 10.1055/s-0033-1335375 10.1055/s-0033-1335375 23605169

[7] Kudo M, Shiina T, Moriyasu F, et al (2013) JSUM ultrasound elastography practice guidelines: liver. Journal of Medical Ultrasonics 40:325–357. 10.1007/s10396-013-0460-5 10.1007/s10396-013-0460-5 27277450

[8] Fujimoto K, Kato M, Kudo M, et al (2013) Novel Image Analysis Method Using Ultrasound Elastography for Noninvasive Evaluation of Hepatic Fibrosis in Patients with Chronic Hepatitis C. Oncology 84:3–12. 10.1159/000345883 10.1159/000345883 23428852

[9] Yada N, Tamaki N, Koizumi Y, et al (2017) Diagnosis of Fibrosis and Activity by a Combined Use of Strain and Shear Wave Imaging in Patients with Liver Disease. Dig Dis 35:515–520. 10.1159/000480140 10.1159/000480140 29040983

[10] Hong H, Li J, Jin Y, et al (2014) Performance of Real-Time Elastography for the Staging of Hepatic Fibrosis: A Meta-Analysis. PLoS ONE 9:e115702. 10.1371/journal.pone.0115702 10.1371/journal.pone.0115702 PMC4277316 25541695

[11] Fang C, Virdee S, Jacob J, et al (2019) Strain elastography for noninvasive assessment of liver fibrosis: A prospective study with histological comparison. Ultrasound 27:262–271. 10.1177/1742271x19862836 10.1177/1742271X19862836 PMC6851718 31762783

[12] Gersak MM, Sorantin E, Windhaber J, et al (2016) The influence of acute physical effort on liver stiffness estimation using Virtual Touch Quantification (VTQ). Preliminary results. Medical Ultrasonography 18:151. 10.11152/mu.2013.2066.182.vtq 10.11152/mu.2013.2066.182.vtq 27239647

[13] Fang C, Lim A, Sidhu PS (2020) Ultrasound-based liver elastography in the assessment of fibrosis. Clin Radiol. 10.1016/j.crad.2020.01.005 10.1016/j.crad.2020.01.005 32067699

[14] Dietrich C, Bamber J, Berzigotti A, et al (2017) EFSUMB Guidelines and Recommendations on the Clinical Use of Liver Ultrasound Elastography, Update 2017 (Long Version). Ultraschall in der Medizin - European Journal of Ultrasound. 10.1055/s-0043-103952 10.1055/s-0043-103952 28407655

[15] Fang C, Jaffer OS, Yusuf GT, et al (2018) Reducing the Number of Measurements in Liver Point Shear-Wave Elastography: Factors that Influence the Number and Reliability of Measurements in Assessment of Liver Fibrosis in Clinical Practice. Radiology 172104. 10.1148/radiol.2018172104 10.1148/radiol.2018172104 29514018

[16] Sporea I, Grădinaru-Taşcău O, Bota S, et al (2013) How many measurements are needed for liver stiffness assessment by 2D-Shear Wave Elastography (2D-SWE) and which value should be used: the mean or median? Med Ultrason 15:268–272 10.11152/mu.2013.2066.154.isp2 24286089

[17] Kim DW, Suh CH, Kim KW, et al (2019) Technical Performance of Two-Dimensional Shear Wave Elastography for Measuring Liver Stiffness: A Systematic Review and Meta-Analysis. Korean J Radiol 20:880. 10.3348/kjr.2018.0812 10.3348/kjr.2018.0812 PMC6536798 31132814

[18] Ferraioli G, Maiocchi L, Lissandrin R, et al (2016) Accuracy of the ElastPQ Technique for the Assessment of Liver Fibrosis in Patients with Chronic Hepatitis C: a “Real Life” Single Center Study. J Gastrointestin Liver Dis 25:331–335 10.15403/jgld.2014.1121.253.epq 27689197

[19] Bota S, Sporea I, Sirli R, et al (2013) Factors which influence the accuracy of acoustic radiation force impulse (ARFI) elastography for the diagnosis of liver fibrosis in patients with chronic hepatitis C. Ultrasound Med Biol 39:407–412. 10.1016/j.ultrasmedbio.2012.09.017 10.1016/j.ultrasmedbio.2012.09.017 23245820

[20] Bota S, Sporea I, Sirli R, et al (2011) Factors that influence the correlation of acoustic radiation force impulse (ARFI) elastography with liver fibrosis. Medical ultrasonography 13:135 21655540

[21] Ferraioli G, De Silvestri A, Lissandrin R, et al (2018) Evaluation of Inter-System Variability in Liver Stiffness Measurements. Ultraschall in der Medizin - European Journal of Ultrasound. 10.1055/s-0043-124184 10.1055/s-0043-124184 29566420

[22] Dietrich CF, Dong Y (2016) Shear wave elastography with a new reliability indicator. J Ultrason 16:281–287. 10.15557/jou.2016.0028 10.15557/JoU.2016.0028 PMC5034022 27679731

[23] Yada N, Sakurai T, Minami T, et al (2015) A Newly Developed Shear Wave Elastography Modality: With a Unique Reliability Index. Oncology 89 Suppl 2:53–59. 10.1159/000440632 10.1159/000440632 26580548

[24] Jeong WK (2015) Liver stiffness measurement using S-Shearwave: initial experience. White Paper, CS201505-S-Shearwave, Samsung Medison

[25] Dong Y, Sirli R, Ferraioli G, et al (2017) Shear wave elastography of the liver - review on normal values. Z Gastroenterol 55:153–166. 10.1055/s-0042-117226 10.1055/s-0042-117226 28192849

[26] Sirli R, Sporea I, Tudora A, et al (2009) Transient elastographic evaluation of subjects without known hepatic pathology: does age change the liver stiffness. J Gastrointestin Liver Dis 18:57–60 19337635

[27] Roulot D, Czernichow S, Clésiau HL, et al (2008) Liver stiffness values in apparently healthy subjects: Influence of gender and metabolic syndrome. Journal of Hepatology 48:606–613. 10.1016/j.jhep.2007.11.020 10.1016/j.jhep.2007.11.020 18222014

[28] Kumar M, Sharma P, Garg H, et al (2011) Transient elastographic evaluation in adult subjects without overt liver disease: influence of alanine aminotransferase levels. J Gastroenterol Hepatol 26:1318–1325. 10.1111/j.1440-1746.2011.06736.x 10.1111/j.1440-1746.2011.06736.x 21443658

[29] Das Kausik, Sarkar Rajib, Ahmed Sk. Mahiuddin, et al (2011) “Normal” liver stiffness measure (LSM) values are higher in both lean and obese individuals: A population‐based study from a developing country. Hepatology 55:584–593. 10.1002/hep.24694 10.1002/hep.24694 21952989

[30] Fung J, Lee C, Chan M, et al (2013) Defining Normal Liver Stiffness Range in a Normal Healthy Chinese Population without Liver Disease. PLoS One 8:. 10.1371/journal.pone.0085067 10.1371/journal.pone.0085067 PMC3873442 24386446

[31] Wong GL-H, Chan HL-Y, Choi PC-L, et al (2013) Association between anthropometric parameters and measurements of liver stiffness by transient elastography. Clin Gastroenterol Hepatol 11:295-302.e1–3. 10.1016/j.cgh.2012.09.025 10.1016/j.cgh.2012.09.025 23022698

[32] Colombo S, Belloli L, Zaccanelli M, et al (2011) Normal liver stiffness and its determinants in healthy blood donors. Dig Liver Dis 43:231–236. 10.1016/j.dld.2010.07.008 10.1016/j.dld.2010.07.008 20817625

[33] Son Chang Young, Kim Seung Up, Han Woong Kyu, et al (2011) Normal liver elasticity values using acoustic radiation force impulse imaging: A prospective study in healthy living liver and kidney donors. Journal of Gastroenterology and Hepatology 27:130–136. 10.1111/j.1440-1746.2011.06814.x 10.1111/j.1440-1746.2011.06814.x 21679249

[34] Kim JE, Lee JY, Kim YJ, et al (2010) Acoustic radiation force impulse elastography for chronic liver disease: comparison with ultrasound-based scores of experienced radiologists, Child-Pugh scores and liver function tests. Ultrasound Med Biol 36:1637–1643. 10.1016/j.ultrasmedbio.2010.07.016 10.1016/j.ultrasmedbio.2010.07.016 20800940

[35] Madhok R, Tapasvi C, Prasad U, et al (2013) Acoustic radiation force impulse imaging of the liver: measurement of the normal mean values of the shearing wave velocity in a healthy liver. J Clin Diagn Res 7:39–42. 10.7860/jcdr/2012/5070.2665 10.7860/JCDR/2012/5070.2665 PMC3576746 23450092

[36] Motosugi U, Ichikawa T, Niitsuma Y, Araki T (2011) Acoustic radiation force impulse elastography of the liver: can fat deposition in the liver affect the measurement of liver stiffness? Jpn J Radiol 29:639–643. 10.1007/s11604-011-0607-5 10.1007/s11604-011-0607-5 21956369

[37] Lee M-J, Kim M-J, Han KH, Yoon CS (2013) Age-related changes in liver, kidney, and spleen stiffness in healthy children measured with acoustic radiation force impulse imaging. Eur J Radiol 82:e290-294. 10.1016/j.ejrad.2013.01.018 10.1016/j.ejrad.2013.01.018 23433651

[38] Ling W, Lu Q, Quan J, et al (2013) Assessment of impact factors on shear wave based liver stiffness measurement. Eur J Radiol 82:335–341. 10.1016/j.ejrad.2012.10.004 10.1016/j.ejrad.2012.10.004 23116805

[39] Sporea I, Bota S, Grădinaru-Taşcău O, et al (2014) Comparative study between two point Shear Wave Elastographic techniques: Acoustic Radiation Force Impulse (ARFI) elastography and ElastPQ. Med Ultrason 16:309–314 10.11152/mu.201.3.2066.164.isp1 25463883

[40] Mulabecirovic A, Mjelle AB, Gilja OH, et al (2018) Liver elasticity in healthy individuals by two novel shear-wave elastography systems—Comparison by age, gender, BMI and number of measurements. PLoS One 13. 10.1371/journal.pone.0203486 10.1371/journal.pone.0203486 PMC6138384 30216377

[41] Yoon JH, Lee JM, Han JK, Cho BI (2014) Shear Wave Elastography for Liver Stiffness Measurement in Clinical Sonographic Examinations: Evaluation of Intraobserver Reproducibility, Technical Failure, and Unreliable Stiffness Measurements. Journal of Ultrasound in Medicine 33:437–447. 10.7863/ultra.33.3.437 10.7863/ultra.33.3.437 24567455

[42] Suh CH, Kim SY, Kim KW, et al (2014) Determination of Normal Hepatic Elasticity by Using Real-time Shear-wave Elastography. Radiology 271:895–900. 10.1148/radiol.14131251 10.1148/radiol.14131251 24555633

[43] Arda K, Ciledag N, Arıbas BK, et al (2013) Quantitative assessment of the elasticity values of liver with shear wave ultrasonographic elastography. Indian J Med Res 137:911–915 PMC3734682 23760376

[44] Huang Z, Zheng J, Zeng J, et al (2014) Normal liver stiffness in healthy adults assessed by real-time shear wave elastography and factors that influence this method. Ultrasound Med Biol 40:2549–2555. 10.1016/j.ultrasmedbio.2014.05.008 10.1016/j.ultrasmedbio.2014.05.008 25282481

[45] Leung VY, Shen J, Wong VW, et al (2013) Quantitative elastography of liver fibrosis and spleen stiffness in chronic hepatitis B carriers: comparison of shear-wave elastography and transient elastography with liver biopsy correlation. Radiology 269:910–918 10.1148/radiol.13130128 23912619

[46] Popescu A, Bota S, Sporea I, et al (2013) The influence of food intake on liver stiffness values assessed by acoustic radiation force impulse elastography-preliminary results. Ultrasound Med Biol 39:579–584. 10.1016/j.ultrasmedbio.2012.11.013 10.1016/j.ultrasmedbio.2012.11.013 23415282

[47] Bota S, Sporea I, Peck-Radosavljevic M, et al (2013) The influence of aminotransferase levels on liver stiffness assessed by Acoustic Radiation Force Impulse Elastography: a retrospective multicentre study. Dig Liver Dis 45:762–768. 10.1016/j.dld.2013.02.008 10.1016/j.dld.2013.02.008 23510533

[48] Goertz RS, Egger C, Neurath MF, Strobel D (2012) Impact of food intake, ultrasound transducer, breathing maneuvers and body position on acoustic radiation force impulse (ARFI) elastometry of the liver. Ultraschall Med 33:380–385. 10.1055/s-0032-1312816 10.1055/s-0032-1312816 22723037

[49] Attia D, Pischke S, Negm AA, et al (2014) Changes in liver stiffness using acoustic radiation force impulse imaging in patients with obstructive cholestasis and cholangitis. Dig Liver Dis 46:625–631. 10.1016/j.dld.2014.02.020 10.1016/j.dld.2014.02.020 24666759

[50] Fraquelli M, Rigamonti C, Casazza G, et al (2007) Reproducibility of transient elastography in the evaluation of liver fibrosis in patients with chronic liver disease. Gut 56:968–973. 10.1136/gut.2006.111302 10.1136/gut.2006.111302 PMC1994385 17255218

[51] Jaffer OS, Lung PFC, Bosanac D, et al (2012) Acoustic radiation force impulse quantification: repeatability of measurements in selected liver segments and influence of age, body mass index and liver capsule-to-box distance. Br J Radiol 85:e858-863. 10.1259/bjr/74797353 10.1259/bjr/74797353 PMC3474007 22763032

[52] Fang C, Konstantatou E, Romanos O, et al (2017) Reproducibility of 2-Dimensional Shear Wave Elastography Assessment of the Liver: A Direct Comparison With Point Shear Wave Elastography in Healthy Volunteers. J Ultrasound Med. 10.7863/ultra.16.07018 10.7863/ultra.16.07018 28370146

[53] Ferraioli G, Tinelli C, Lissandrin R, et al (2014) Point shear wave elastography method for assessing liver stiffness. World J Gastroenterol 20:4787–4796. 10.3748/wjg.v20.i16.4787 10.3748/wjg.v20.i16.4787 PMC4000517 24782633

[54] Bota S, Sporea I, Sirli R, et al (2012) Intra- and interoperator reproducibility of acoustic radiation force impulse (ARFI) elastography--preliminary results. Ultrasound Med Biol 38:1103–1108. 10.1016/j.ultrasmedbio.2012.02.032 10.1016/j.ultrasmedbio.2012.02.032 22579536

[55] Ferraioli G, Tinelli C, Zicchetti M, et al (2012) Reproducibility of real-time shear wave elastography in the evaluation of liver elasticity. European Journal of Radiology 81:3102–3106. 10.1016/j.ejrad.2012.05.030 10.1016/j.ejrad.2012.05.030 22749107

[56] Piccinino F, Sagnelli E, Pasquale G, Giusti G (1986) Complications following percutaneous liver biopsy. A multicentre retrospective study on 68,276 biopsies. J Hepatol 2:165–173 10.1016/s0168-8278(86)80075-7 3958472

[57] McGill DB, Rakela J, Zinsmeister AR, Ott BJ (1990) A 21-year experience with major hemorrhage after percutaneous liver biopsy. Gastroenterology 99:1396–1400 10.1016/0016-5085(90)91167-5 2101588

[58] Bedossa P, Dargère D, Paradis V (2003) Sampling variability of liver fibrosis in chronic hepatitis C. Hepatology 38:1449–1457. 10.1016/j.hep.2003.09.022 10.1016/j.hep.2003.09.022 14647056

[59] Persico M, Palmentieri B, Vecchione R, et al (2002) Diagnosis of chronic liver disease: reproducibility and validation of liver biopsy. The American Journal of Gastroenterology 97:491–492. 10.1111/j.1572-0241.2002.05507.x 10.1111/j.1572-0241.2002.05507.x 11866299

[60] European Association for Study of Liver, Asociacion Latinoamericana para el Estudio del Higado (2015) EASL-ALEH Clinical Practice Guidelines: Non-invasive tests for evaluation of liver disease severity and prognosis. J Hepatol 63:237–264. 10.1016/j.jhep.2015.04.006 10.1016/j.jhep.2015.04.006 25911335

[61] Virtual Touch Quantification to diagnose and monitor liver fibrosis in chronic hepatitis B and C | Guidance and guidelines | NICE. https://www.nice.org.uk/guidance/mtg27. Accessed 13 Jan 2019

[62] Xu X, Su Y, Song R, et al (2015) Performance of transient elastography assessing fibrosis of single hepatitis B virus infection: a systematic review and meta-analysis of a diagnostic test. Hepatol Int 9:558–566. 10.1007/s12072-015-9643-z 10.1007/s12072-015-9643-z 26187292

[63] Chon YE, Choi EH, Song KJ, et al (2012) Performance of Transient Elastography for the Staging of Liver Fibrosis in Patients with Chronic Hepatitis B: A Meta-Analysis. PLOS ONE 7:e44930. 10.1371/journal.pone.0044930 10.1371/journal.pone.0044930 PMC3458028 23049764

[64] Li Y, Huang Y-S, Wang Z-Z, et al (2016) Systematic review with meta-analysis: the diagnostic accuracy of transient elastography for the staging of liver fibrosis in patients with chronic hepatitis B. Aliment Pharmacol Ther 43:458–469. 10.1111/apt.13488 10.1111/apt.13488 26669632

[65] Hu X, Qiu L, Liu D, Qian L (2017) Acoustic Radiation Force Impulse (ARFI) Elastography for non‑invasive evaluation of hepatic fibrosis in chronic hepatitis B and C patients: a systematic review and meta-analysis. Med Ultrason 19:23–31 10.11152/mu-942 28180193

[66] Fu J, Wu B, Wu H, et al (2020) Accuracy of real-time shear wave elastography in staging hepatic fibrosis: a meta-analysis. BMC Med Imaging 20:16. 10.1186/s12880-020-0414-5 10.1186/s12880-020-0414-5 PMC7014748 32046659

[67] Lee SM, Lee JM, Kang H-J, et al (2017) Liver fibrosis staging with a new 2D-shear wave elastography using comb-push technique: Applicability, reproducibility, and diagnostic performance. PLOS ONE 12:e0177264. 10.1371/journal.pone.0177264 10.1371/journal.pone.0177264 PMC5433696 28510583

[68] Jiang W, Huang S, Teng H, et al (2018) Diagnostic accuracy of point shear wave elastography and transient elastography for staging hepatic fibrosis in patients with non-alcoholic fatty liver disease: a meta-analysis. BMJ Open 8:e021787. 10.1136/bmjopen-2018-021787 10.1136/bmjopen-2018-021787 PMC6112406 30139901

[69] Herrmann E, de Lédinghen V, Cassinotto C, et al (2018) Assessment of biopsy‐proven liver fibrosis by two‐dimensional shear wave elastography: An individual patient data‐based meta‐analysis. Hepatology 67:260–272. 10.1002/hep.29179 10.1002/hep.29179 PMC5765493 28370257

[70] Cassinotto C, Lapuyade B, Mouries A, et al (2014) Non-invasive assessment of liver fibrosis with impulse elastography: comparison of Supersonic Shear Imaging with ARFI and FibroScan^®^. J Hepatol 61:550–557. 10.1016/j.jhep.2014.04.044 10.1016/j.jhep.2014.04.044 24815876

[71] Foncea CG, Popescu A, Lupusoru R, et al (2020) Comparative study between pSWE and 2D-SWE techniques integrated in the same ultrasound machine, with Transient Elastography as the reference method. Med Ultrason 22:13–19. 10.11152/mu-2179 10.11152/mu-2179 32096782

[72] Schellhaas B, Strobel D, Wildner D, et al (2017) Two-dimensional shear-wave elastography: a new method comparable to acoustic radiation force impulse imaging? Eur J Gastroenterol Hepatol. 10.1097/MEG.0000000000000846 10.1097/MEG.0000000000000846 28118179

[73] Nierhoff J, Chávez Ortiz AA, Herrmann E, et al (2013) The efficiency of acoustic radiation force impulse imaging for the staging of liver fibrosis: a meta-analysis. European Radiology 23:3040–3053. 10.1007/s00330-013-2927-6 10.1007/s00330-013-2927-6 23801420

[74] Bota S, Herkner H, Sporea I, et al (2013) Meta-analysis: ARFI elastography versus transient elastography for the evaluation of liver fibrosis. Liver International 33:1138–1147. 10.1111/liv.12240 10.1111/liv.12240 23859217

[75] Hall TJ, Milkowski A, Garra B, et al (2013) RSNA/QIBA: Shear wave speed as a biomarker for liver fibrosis staging. In: Ultrasonics Symposium (IUS), 2013 IEEE International. IEEE, pp 397–400

[76] Dillman JR, Chen S, Davenport MS, et al (2015) Superficial Ultrasound Shear Wave Speed Measurements in Soft and Hard Elasticity Phantoms: Repeatability and Reproducibility Using Two Different Ultrasound Systems. Pediatr Radiol 45:376–385. 10.1007/s00247-014-3150-6 10.1007/s00247-014-3150-6 PMC4346477 25249389

[77] Shin HJ, Kim M-J, Kim HY, et al (2016) Comparison of shear wave velocities on ultrasound elastography between different machines, transducers, and acquisition depths: a phantom study. Eur Radiol 26:3361–3367. 10.1007/s00330-016-4212-y 10.1007/s00330-016-4212-y 26815368

[78] European Association for the Study of the Liver. Electronic address: easloffice@easloffice.eu, European Association for the Study of the Liver (2018) EASL Recommendations on Treatment of Hepatitis C 2018. J Hepatol 69:461–511. 10.1016/j.jhep.2018.03.026 10.1016/j.jhep.2018.03.026 29650333

[79] Jung YK, Yim HJ (2017) Reversal of liver cirrhosis: current evidence and expectations. The Korean Journal of Internal Medicine 32:213–228. 10.3904/kjim.2016.268 10.3904/kjim.2016.268 PMC5339475 28171717

[80] Knop V, Hoppe D, Welzel T, et al (2016) Regression of fibrosis and portal hypertension in HCV-associated cirrhosis and sustained virologic response after interferon-free antiviral therapy. J Viral Hepat 23:994–1002. 10.1111/jvh.12578 10.1111/jvh.12578 27500382

[81] D’Ambrosio R, Aghemo A, Fraquelli M, et al (2013) The diagnostic accuracy of Fibroscan^®^ for cirrhosis is influenced by liver morphometry in HCV patients with a sustained virological response. Journal of Hepatology 59:251–256. 10.1016/j.jhep.2013.03.013 10.1016/j.jhep.2013.03.013 23528378

[82] Younossi ZM, Golabi P, de Avila L, et al (2019) The global epidemiology of NAFLD and NASH in patients with type 2 diabetes: A systematic review and meta-analysis. J Hepatol 71:793–801. 10.1016/j.jhep.2019.06.021 10.1016/j.jhep.2019.06.021 31279902

[83] Perumpail BJ, Khan MA, Yoo ER, et al (2017) Clinical epidemiology and disease burden of nonalcoholic fatty liver disease. World J Gastroenterol 23:8263–8276. 10.3748/wjg.v23.i47.8263 10.3748/wjg.v23.i47.8263 PMC5743497 29307986

[84] Pais R, Barritt AS, Calmus Y, et al (2016) NAFLD and liver transplantation: Current burden and expected challenges. J Hepatol 65:1245–1257. 10.1016/j.jhep.2016.07.033 10.1016/j.jhep.2016.07.033 PMC5326676 27486010

[85] Ekstedt M, Hagström H, Nasr P, et al (2015) Fibrosis stage is the strongest predictor for disease-specific mortality in NAFLD after up to 33 years of follow-up. Hepatology 61:1547–1554. 10.1002/hep.27368 10.1002/hep.27368 25125077

[86] EASL-EASD-EASO Clinical Practice Guidelines for the management of non-alcoholic fatty liver disease (2016). J Hepatol: 64(6): 1388-402. 10.1016/j.jhep.2015.11.004. 10.1016/j.jhep.2015.11.004 27062661

[87] Xiao G, Zhu S, Xiao X, et al (2017) Comparison of laboratory tests, ultrasound, or magnetic resonance elastography to detect fibrosis in patients with nonalcoholic fatty liver disease: A meta-analysis. Hepatology 66:1486–1501. 10.1002/hep.29302 10.1002/hep.29302 28586172

[88] Friedrich-Rust M, Hadji-Hosseini H, Kriener S, et al (2010) Transient elastography with a new probe for obese patients for non-invasive staging of non-alcoholic steatohepatitis. Eur Radiol 20:2390–2396. 10.1007/s00330-010-1820-9 10.1007/s00330-010-1820-9 20526777

[89] Yoneda M, Suzuki K, Kato S, et al (2010) Nonalcoholic fatty liver disease: US-based acoustic radiation force impulse elastography. Radiology 256:640–647. 10.1148/radiol.10091662 10.1148/radiol.10091662 20529989

[90] Palmeri ML, Wang MH, Rouze NC, et al (2011) Noninvasive evaluation of hepatic fibrosis using acoustic radiation force-based shear stiffness in patients with nonalcoholic fatty liver disease. J Hepatol 55:666–672. 10.1016/j.jhep.2010.12.019 10.1016/j.jhep.2010.12.019 PMC3092839 21256907

[91] Fierbinteanu Braticevici C, Sporea I, Panaitescu E, Tribus L (2013) Value of acoustic radiation force impulse imaging elastography for non-invasive evaluation of patients with nonalcoholic fatty liver disease. Ultrasound Med Biol 39:1942–1950. 10.1016/j.ultrasmedbio.2013.04.019 10.1016/j.ultrasmedbio.2013.04.019 23932277

[92] Mueller S, Millonig G, Sarovska L, et al (2010) Increased liver stiffness in alcoholic liver disease: Differentiating fibrosis from steatohepatitis. World J Gastroenterol 16:966–972. 10.3748/wjg.v16.i8.966 10.3748/wjg.v16.i8.966 PMC2828601 20180235

[93] Thiele M, Detlefsen S, Sevelsted Møller L, et al (2016) Transient and 2-Dimensional Shear-Wave Elastography Provide Comparable Assessment of Alcoholic Liver Fibrosis and Cirrhosis. Gastroenterology 150:123–133. 10.1053/j.gastro.2015.09.040 10.1053/j.gastro.2015.09.040 26435270

[94] Nahon P, Kettaneh A, Tengher-Barna I, et al (2008) Assessment of liver fibrosis using transient elastography in patients with alcoholic liver disease. Journal of Hepatology 49:1062–1068. 10.1016/j.jhep.2008.08.011 10.1016/j.jhep.2008.08.011 18930329

[95] Nguyen‐Khac E, Chatelain D, Tramier B, et al (2008) Assessment of asymptomatic liver fibrosis in alcoholic patients using fibroscan: prospective comparison with seven non-invasive laboratory tests. Alimentary Pharmacology & Therapeutics 28:1188–1198. 10.1111/j.1365-2036.2008.03831.x 10.1111/j.1365-2036.2008.03831.x 18705692

[96] Janssens F, de Suray N, Piessevaux H, et al (2010) Can transient elastography replace liver histology for determination of advanced fibrosis in alcoholic patients: a real-life study. J Clin Gastroenterol 44:575–582. 10.1097/mcg.0b013e3181cb4216 10.1097/MCG.0b013e3181cb4216 20104185

[97] Bardou-Jacquet E, Legros L, Soro D, et al (2013) Effect of alcohol consumption on liver stiffness measured by transient elastography. World J Gastroenterol 19:516–522. 10.3748/wjg.v19.i4.516 10.3748/wjg.v19.i4.516 PMC3558575 23382630

[98] Snowdon VK, Guha N, Fallowfield JA (2012) Noninvasive evaluation of portal hypertension: emerging tools and techniques. Int J Hepatol 2012:691089. 10.1155/2012/691089 10.1155/2012/691089 PMC3376538 22720166

[99] Bosch J, Abraldes JG, Berzigotti A, García-Pagan JC (2009) The clinical use of HVPG measurements in chronic liver disease. Nat Rev Gastroenterol Hepatol 6:573–582. 10.1038/nrgastro.2009.149 10.1038/nrgastro.2009.149 19724251

[100] Shi K-Q, Fan Y-C, Pan Z-Z, et al (2013) Transient elastography: a meta-analysis of diagnostic accuracy in evaluation of portal hypertension in chronic liver disease. Liver Int 33:62–71. 10.1111/liv.12003 10.1111/liv.12003 22973991

[101] Thiele M, Hugger MB, Kim Y, et al 2D shear wave liver elastography by aixplorer to detect portal hypertension in cirrhosis: an individual patient data meta-analysis. Liver International n/a: 10.1111/liv.14439 10.1111/liv.14439 32180327

[102] Augustin S, Pons M, Maurice JB, et al (2017) Expanding the Baveno VI criteria for the screening of varices in patients with compensated advanced chronic liver disease. Hepatology. 10.1002/hep.29363 10.1002/hep.29363 28696510

[103] LeCun Y, Bengio Y, Hinton G (2015) Deep learning. Nature 521:436–444. 10.1038/nature14539 10.1038/nature14539 26017442

[104] Litjens G, Kooi T, Bejnordi BE, et al (2017) A survey on deep learning in medical image analysis. Medical Image Analysis 42:60–88. 10.1016/j.media.2017.07.005 10.1016/j.media.2017.07.005 28778026

[105] Wang K, Lu X, Zhou H, et al (2019) Deep learning Radiomics of shear wave elastography significantly improved diagnostic performance for assessing liver fibrosis in chronic hepatitis B: a prospective multicentre study. Gut 68:729–741. 10.1136/gutjnl-2018-316204 10.1136/gutjnl-2018-316204 PMC6580779 29730602

[106] Gatos I, Tsantis S, Spiliopoulos S, et al (2019) Temporal stability assessment in shear wave elasticity images validated by deep learning neural network for chronic liver disease fibrosis stage assessment. Med Phys 46:2298–2309. 10.1002/mp.13521 10.1002/mp.13521 30929260

